# An Ishmaelite

**Published:** 1888-07-14

**Authors:** Leslie Keith

**Affiliations:** Author of "The Chilcotes," "Uncle Bob's Niece," "East and West," etc.


### AN ISHMAELITE.

BY LESLIE KEITH,

Author of "The Chilcotes," "Uncle Bob's Niece," "East and West," etc.

(*Continued from p. 235.*)

### CHAPTER XIV.

ARTHUR felt himself somewhat of a captive led in chains when he accompanied Ella to Norfolk. He could scarcely be said to be going as her protector, since Ella required no dragon. She was the pink of propriety and self-possession, and he would have been a bold man who had dared to address a disrespectful word to her.

Nor did she require any of those little attentive cares which it would have given him pleasure to bestow. Arthur had very kindly feelings for women and children, and liked to render them small services. But Ella asked nothing of his hands. Her parasol and wraps were strapped with the most precise neatness; she had furnished herself with a mildly proper novel, and with a luncheon basket fitted for two, that left nothing to be desired.

"You don't want a cavalier, Ella. You could go round the world alone. What! have you actually got your own ticket?"

"Yes, I like to do things for myself. Every woman ought to be able to travel alone."

"I thought it was one of the things Mrs. Grundy objected to."

"I mean, when one must go alone. Of course, it is much better that you should go with me. It is what papa and mamma will expect."

"So I am being offered up to the proprieties, after all," said Arthur, with a laugh that was not quite spontaneous. "And I suppose I must not smoke? Take your book, my dear; don't mind me—you may consider me part of your luggage; I will behave as quietly as if I were one of your trunks or band-boxes.

Ella never understood him when he was in this bitter mood, but she had a lady-like way of ignoring his sarcasms.

"I don't particularly want to read," she said; and she put down her book and folded her hands, and looked calmly out at the flying landscape.

They were alone in the carriage. It flashed across Arthur all at once that they might have been upon their wedding journey. Ella's neat, cool summer draperies might very well pass for the travelling gear of a bride; but where was the bridal joy her face ought to have worn? and where was the responsive gladness in his own heart? He shrank back before this parody—this travesty, as it seemed to him, of married bliss. He was abashed, too, before the glimpse he all at once had of his own feelings.

"Will it be like this always?" he thought; "shall we be together, living side by side, but, like two parallel lines, with never a meeting-place?"

At the station cousin Fanny was awaiting them in the one-horse fly, hired for solemn occasions. She gave Arthur a cool, firm cheek to kiss, by which he understood that he was an elected member of the family. He was glad to get upon the box beside the shabby driver, and to take out his pipe, and smoke away some of his discontent.

The country lanes were fresh and sweet after the used-up air of London; and yet his thoughts were travelling backwards with the up-train, whose smoke left a stain upon the blue sky. "How hot and airless it must be in Gillespie Street," he was thinking; "do they never go away." Delia had told him that she sometimes went for a week with her mother and aunt to Margate or Ramsgate. "How she would like those shady lanes and liberal hedgerows," he thought, "and those wild-flowers." When it came to thinking such thoughts as these, and to wishing, as he did, that he could give her this pleasure, Arthur had some cause to distrust himself.

At the Rectory the other girls were waiting to welcome the travellers. He scarcely knew those younger cousins, but he was struck with the strong family look that they had conscientiously handed down from the eldest to the youngest. They were all tall and rather good-looking, with big, light-blue eyes, flaxen hair smoothly braided, and cool, firm, pink cheeks.

Arthur kissed them all in turn, but he found it an unexciting business. The rector presently shuffled out on the gravel in a pair of carpet slippers, his blue spectacles shining like second eyes high up on his forehead. He shook hands with Arthur heartily, and said—

"How do you do, Mr. Penfold? Very glad to see you."

"It is not Mr. Penfold," said cousin Fanny sharply; "it is Arthur Eastgate, Joseph's nephew."

"It is such a long time since Arthur was here; he can scarcely expect papa to remember him," said Ella, going up and dutifully greeting her father. Arthur watched the meeting, and it seemed to him as if the bewildered rector had had time to forget his elder daughter also.

"Oh—ah! it is Ella—to be sure," he said, when the meaning of her caress at last dawned upon him. "And Arthur—Arthur Eastgate—yes, yes, I remember."

"Mr. Coates is always in a dream," said his wife, with an impatient sigh. "Come, Arthur, I will show you your room. I have given you Jim's room, because the spare on will be wanted to-morrow. But I don't treat you like a stranger; you are one of the family now."

It was part of the privilege of being adopted, as he found, to be consigned to very narrow quarters, and to be put on short commons in the matter of towels and soap. Jim seemed to have inherited the discarded lumber of various other rooms in the way of furniture, since no article matched with its neighbour, and all were more or less infirm. But Arthur's fastidiousness did not go out in the shape of æsthetic longings, and he only spared a passing wonder, as he hastily brushed off the signs of travel, for whom the glories of the spare chamber were reserved.

Cousin Fanny had left him with an admonition not to waste time on a toilet.

"We dine at two on Saturdays," she said. "I thought you would not like us to make any difference for you."

Being thus charged, Arthur was so expeditious that he found himself the first to arrive in the drawing-room. Presently, however, the youngest of the cousins, a long-legged, awkward girl of thirteen, joined him, and from her artless chatter he soon was in possession of the secret concerning the expected guest.

"How quick you have been, Cousin Arthur," she said. "Fanny and Clara are helping Ella to unpack, but they wouldn't let me stay. Clara is to have one of Ella's dresses to wear to-morrow, and she is to try it on to-day, in case it doesn't fit."

"What is going to happen to-morrow, Lettie? Do you always wear grand frocks on Sunday?"

"Oh, no. We're to have on our best frocks, but only Clara is to be at dinner, because Mr. Penfold is coming, and dinner is to be late."

"And who is Mr. Penfold?"

"Oh," said Lettie, "he is very rich," as if in saying that she had said everything. "He's rather old, at least I think so; but mamma says he's only forty. Forty is pretty old, isn't it, Arthur?"

"Pretty old to fourteen, Lettie, but I daresay you and I will think it quite young when we get to it. And what is this elderly Mr. Penfold coming for?"

"He's coming for dinner," said the literal Miss Lettie, "and to stop all night. He is to have the spare room; mamma has been making new curtains for it, and she has put the sofa in it out of her own room. Mr. Penfold is coming to look at a place here that he thinks he will buy, if he likes it."

Arthur burst out laughing, and laughed so long and heartily that Lettie looked at him with reproach.

"Why do you laugh?" she said. She shared the family lack of humour. "Is it so queer that he should want to buy a place? I think it is very nice to be rich enough to buy anything you like."

"Oh, very nice," said Arthur, recovering his gravity. "Let us hope that Mr. Penfold will sleep well behind the new curtains, and find the sofa comfortable."

"It's a very comfortable sofa," said Lettie, with a curious little parody of her elder sister's sedate manner. "I sleep on it sometimes when the boys are all at home, and the house is full. You ought to have had the spare room, Arthur, but mamma said you wouldn't mind using Jim's, because you are one of the family."

"Yes," said Arthur, laughing again. "And perhaps it will be Mr. Penfold's turn to be put in Jim's room some day, when—when another elderly gentleman comes in search of a country mansion."

When Clara came down to dinner, wearing a ribbon that he recognised as Ella's property, he felt himself to be in complete possession of the family tactics, and he could scarcely spare his sarcasms.

And yet cousin Fanny was only doing what many another anxious mother thinks it her first duty to do, in finding husbands and homes for her daughters. Ella was off her hands, and why should not Clara have her chance, and why should match-making seem so much more of a crime in Norfolk than in London?

If Arthur's mood had not been so savage, he might have even found something heroic in the mother's efforts to provide a better lot for the young ones than had fallen to her own share: she exacted nothing for herself, if she pushed and schemed for her children; her own dress was the shabbiest and poorest in the household; it was patched and turned, that Clara or Minnie might not go without some coveted bit of finery; if there was cold meat, it was she who ate it, while the chop or steak was reserved for papa. How she toiled and saved, and planned and scraped, to save a little fund for the children, no one ever knew; perhaps she had not always been so persistent, so bold in asking favours, so ready to grasp every little advantage: it may have been necessity that gave her that hard, keen look, and that self-asserting manner that Arthur found so intolerable.

He preferred c usin Fanny's husband a thousand-fold, and slunk away to the study after dinner, to smoke with the peaceful, lazy, absent vicar, who sat all day surrounded with books which he never read, though he was understood to be always deeply absorbed in studious meditation.

The library had been inherited from his father, who was vicar here before him; and the sermons were inherited too, as Arthur discovered to his amusement when he found the pile in faded ink from which to-morrow's discourse was to be selected, lying under the *Norfolk Herald*. He glanced at the theology on the shelves, and found the dust of months reposing on the old divines; indeed, they were never disturbed except when Mrs. Coates or her daughters made a raid on them at periodical epochs of cleaning.

But if the vicar did not study, neither did he bother his guest with inconsequent chatter; he was the most silent man it had ever been Arthur's lot to meet, and perhaps the most easily decipherable. He had an air of meditative patience and of unshaken placidity and resignation to let himself be done for, which was certainly a peculiarly happy disposition for a man with a wife and six daughters all famed for their management and common-sense. It was perhaps the conviction that his future father-in-law had not much common-sense that drew Arthur to him and made him find something very sociable in his silence.

He was routed from this haven at supper time, and when the meal was over, cousin Fanny took him in hand. This excellent woman had a way of going directly at any subject without any prelude, which her husband's vagueness had perhaps helped her to acquire. She marched Arthur round the garden.

"Arthur," she said, "I understand from Ella that you are in constant communication with Frederick Eastgate."

"Yes," said Arthur, suffering a little shock," I see him often, but not often enough for my wishes."

"Perhaps, however, too often for your good. I think it an immense pity you did not insist on his going abroad. He may embarrass you seriously. A young man about to marry and settle down ought not to have any equivocal ties."

Arthur turned upon her with a flash of his blue eyes.

"You are speaking of my cousin—my brother, I may say," he said. "If I could have persuaded him to go abroad with me, I should not have been here to-day to defend him. I should have thought it scarcely necessary to tell you that I will never give him up, and so far from freeing myself from him, I will do my utmost to get him to share any home I may have."

"Not while Ella is mistress of it."

"You are a clergyman's wife, cousin Fanny, and I suppose you preach repentance to village sinners. Are the prodigals never allowed to come back? are they to do all their repenting among the swine, and never to be received in the old home again?"

In her way she was a good woman, and she reddened dully under his thrusts. She did not resent his anger.

"Perhaps you are right, Arthur," she said with unusual hesitation. "And if I were differently circumstanced—if I hadn't my girls to think of,—but what may be quite a duty for you, need be none for Ella; you must not compromise my daughter, or—or lead her to quarrel with your uncle. A young wife has to be very careful in the acquaintances she makes, and Ella would shrink from association with your cousin. It would be painful to her. Richard Bellynge would not permit such an acquaintance for Nellie, and I must be equally exacting for my child."

"No," said Arthur with a sigh; "I see I must give up my idea of having Fred to live with us."

"It would be quite out of the question."

"Well, I shall not urge Ella, or influence her at all. I have tried it," he admitted, with rather bitter frankness; "and I haven't succeeded. Ella doesn't always adopt my point of view."

"She has a very practical way of looking at things; you would do well to be guided by her judgment."

"Oh, yes," said Arthur, making haste to quit this subject, "she is certainly practical."

It seemed to him that the family traits were sufficiently in evidence without requiring illustration.

"I am going to leave her with you," he said, breaking his intentions as lightly as possible; "I'm off on Monday—"

"Are you going to leave us on Monday?"

"Uncle Joseph is alone, and you've another guest, you know."

It was a happy inspiration to introduce the future occupant of the spare room. It immediately recalled to cousin Fanny all those multifarious cares that were necessary to the due honouring of Mr. Penfold, and what with the Saturday night's weight on her mind, of sheets to be aired, to-morrow's dinner and the succeeding morning's breakfast, the hymns and the vicar's clean surplice, he escaped with an easily borne reprimand.

When cousin Fanny had rushed off to test the hymns on the squeaking little harmonium, Arthur sauntered about alone in the dewy garden. He could hear the girls' voices coming from the room where the unpacking and examining of Ella's finery was still going on, and crossing them their mother's conscientious pounding of Mozart's *Ave Verum* arranged as a voluntary.

Presently Lettie's long legs came in a kind of weak-kneed trot across the grass. She was shut out of that paradise of new clothes upstairs, and looked on Arthur as a fellow exile.

"Lettie," he said, "are there any wild-flowers about, any cowslips or primroses? I saw lots as we came—"

"Oh, Arthur! There are no primroses in August."

"Well, dog-roses, or—or flowers, anyhow. Could you tear yourself away from Mr. Penfold, and help me to get some on Monday morning."

"Oh, yes," said Lettie, rather flattered to be thus appealed to, "there are some foxgloves at the edge of the wood, and there is briony in the hedges; I'll show you where they grow."

"All right, it's a bargain, Lettie."

"What do you want them for, Arthur?"

Arthur smiled.

"I want to take a little bit of the country back to London with me, Miss Inquisitive."

"To remember Ella by?" asked this inconvenient maiden. But this time she got no reply.

*(To be continued.)*

